# The application of enhanced recovery after surgery and negative-pressure wound therapy in the perioperative period of elderly patients with colorectal cancer

**DOI:** 10.1186/s12893-021-01331-y

**Published:** 2021-09-06

**Authors:** Han-rong Liu, Ping Yang, Song Han, Yu Zhang, Hui-yin Zhu

**Affiliations:** 1 Department of General Surgery, Shanghai Fourth People’s Hospital, Affiliated to Tongji University School of Medicine, No.1297 sanmen Road, Hongkou District, Shanghai, China; 2 Department of Internal Medicine, Shanghai Pengpu Community Health Service Center, Shanghai, China

**Keywords:** Enhanced recovery after surgery, Colorectal cancer, The elderly patient, Negative-pressure wound therapy

## Abstract

**Objective:**

To Explore the perioperative application of enhanced recovery after surgery (ERAS) and negative-pressure wound therapy in the elderly patients with colorectal cancer.

**Methods:**

A retrospective clinical data were studied in the patients with colorectal cancer in Department of General Surgery in Shanghai Fourth People,s Hospital (from March, 2017 to March, 2019), One hundred and fifty patients with undergoing radical surgery for colorectal cancer were divided into two groups: ERAS group (n = 76 cases, accepting ERAS management) and Conventional treatment(CT) group (n = 74 cases, accepting traditional treatment), Bleeding in operation, the time of postoperative anal flatus, number of wound dressing changing, time of wound healing, the length of postoperative hospital stay, readmission rate, postoperative complication, were compared between the two groups.

**Results:**

ERAS was associated with less bleeding in operation, less Wound fat liquefaction, less wound dressing changing, less time of wound healing, less time of postoperative anal flatus compare to CT group (*P* < 0.05); anastomotic fistula, readmission rate is similar in two groups (*P* > 0.05).

**Conclusion:**

The modified ERAS can be safely applied to the perioperative period of elderly colorectal cancer patients and promote recovery; negative-pressure wound therapy is helpful for wound healing and promoting rehabilitation.

## Introduction

An increasing number of elderly patients is subjected to colorectal surgery. In particular colorectal carcinoma has a peak of incidence in the seventh and eight decade of life [1, 2]. In 2016, the estimated number of new cases of rectal cancer was 39,220 in the United States. Although the incidence and death rates of colorectal cancer declined by 3% per year from 2003 to 2012, colorectal cancer remains the second leading cause of death in men ages 60–79 and the third leading cause of death in men over 80 years old and in women over 60 years old [3].

In the past 30 years, the incidence of colorectal cancer in China has been increasing year by year, especially in economically developed areas. Take Shanghai, China as an example, the incidence of colorectal cancer has changed significantly. In 1962, colorectal cancer was only the 7th most common malignant tumor in Shanghai, but in 2003, it has become the second most common malignant tumor. From 1978 to 1982, the incidence of colorectal cancer was 19.9 per 100,000 males and 19.1 per 100,000 females. From 1988 to 1992, the incidence of colorectal cancer was 27.0 per 100,000 males and 26.6 per 100,000 females. In 1997, the incidence of colorectal cancer was 37.2 per 100,000 males and 36.5 per 100,000 females respectively. In 2003, 51.2% of the patients with colorectal cancer in Shanghai urban area were > 70 years old. With the continuous emergence of aging city, colorectal cancer in the elderly will become a key research topic.

The data to guide treatment of elderly patients with rectal cancer are sparse since the elderly population has been underrepresented in prospective clinical trials involving colorectal cancer [4]. A population-based study in rectal cancer showed that age was the strongest determinant of treatment and that with advancing age there was a decline in the proportion of patients receiving standard of care adjuvant therapy even after adjusting for co-morbidities [5].

There is much anemia, hypoproteinemia and internal medicine diseases in the gerontal patients of colorectal cancer, Tissue healing is poor, It is a problem how to promote the surgical endurance and recovery after surgery of the gerontal patients, It is a problem that surgeons face all the time. Enhanced recovery after surgery (ERAS) is a kind of concept that reduces surgical stress and complications to apply various proven methods in perioperative period and accelerate the recovery of patients after surgery. Since being first described by Kehlet and Mogensen [6], there has been widespread adoption by colorectal surgeons of enhanced recovery after surgery (ERAS) programmes [6–9]. ERAS consists of multimodal components including shorter fasting times, carbohydrate preloading, preoperative counselling, appropriate fluid therapy, early initiation of oral diet and early mobilisation, which aimed to standardise and subsequently optimise postoperative care [10, 11].

How to recover quickly in elderly patients with colorectal cancer in perioperative period, How to make the concept of rapid recovery better reflected in the elderly patients, We apply modified ERAS therapy in part elderly patients(> the 70-year-old) with colorectal cancer in General Surgery of Shanghai Fourth People’s Hospital, It is compared with the conventional treatment patients in same period, conventional treatment is the treatment method before ERAS, without the concept and method of alleviating stress and complications to promote postoperative recovery of patients, To analyze the effect of the concept and measures of ERAS on postoperative recovery, the study was approved by the Ethics Committee of Shanghai Fourth People’s Hospital.

## Methods

### Patients

One hundred and fifty elderly patients with colorectal tumors in General Surgery of Shanghai Forth People’s hospital from March 2017 to March 2019 were reviewed, They were operated by general surgeons and divided into two groups: Enhanced recovery after surgery group (ERAS) seventy-six patients, Conventional therapy group (CT) seventy-four patients, The specific surgical methods are shown in Table 1.

**Table 1:** Demographic and clinical characteristics for the ERAS group and CT group

	ERAS	CT
Age(years)	78.04 ± 5.74	78.74 ± 6.56
Male:Female	34:42	40:34
Tumor location		
Right colon and flexure	30	22
Transverse colon	2	12
Left colon and left flexure	10	8
Rectum	18	6
Sigmoid	16	26
Scope of surgery		
Right hemicolectomy	28	34
Left hemicolectomy	10	6
Anterior/Low anterior resection	12	8
Abdominoperineal resection	4	0
Radical resection of sigmoid carcinoma	18	24
Other operations	4	2
Preoperative Comorbidities		
Preoperative anemia	15	12
Preoperative hypoproteinemia	6	10
Coronary heart disease	5	13
Diabetes mellitus	18	8
Hypertensive disease	33	16
Cerebral infarction	5	7
Preoperative nutritional support	19	18

### Inclusion criteria

Patients with colorectal cancer were definitely diagnosed by fiberoptic electron colonoscopy and histopathology, Tumor TNM staging of I–III period, over 70 years of age, no surgical contraindications。exclusion criteria: Heart, lung, liver and kidney severe dysfunction; The basic data of the two groups are shown in Table 1, Most of the patients in both groups had different degrees of hypertensive, diabetic and heart diseases, There was no significant difference between the two groups in gender, age and surgical methods.

### Perioperative period process mode

The modified ERAS protocol are applied the patients of ERAS Group, They include informing treatment procedures, Informing the operation mode and recovery time before operation, modest comfort, soothing the patient's nerves before operation, accurate operation reducing trauma and blood loss, encouraging moderate activity postoperation, Drinking water 6 h after surgery, actively cooperating during treatment, Adopting a series of quick rehabilitation measures such as relieving pain (multi-mode analgesia reducing the dose of morphine) and reducing stress, no gastric tube, negative-pressure wound therapy (the wound was drained by a subcutaneous drainage tube with multi-side holes); In conventional treatment group we used conventional treatment, Specific methods are shown in Table 2, Both groups used multi-mode analgesia reducing the dose of morphine postoperatively, Some patients in the two groups had malnutrition、anemia before operation and were given appropriate nutritional support and minor transfusion correction, Both groups were elderly patients, Perioperative fluid intake was controlled, (< 30 ml/Kg body weight/day).

**Table 2:** ERAS group and CT group perioperative management

Perioperative management	ERAS group	CT group
Perioperative publicity and education	Systematic and purposeful	General publicity and education
Preoperative intestinal preparation	Preparation of half compound polyethylene glycol electrolyte powder	Routine preparation of compound polyethylene glycol electrolyte powder
Preoperative oral antibiotic bowel preparation	Gentamicin 8 mg, metronidazole 400 mg (1, 5, 9 PM before surgery)	Gentamicin 8 mg, metronidazole 400 mg (1, 5, 9 PM before surgery)
Preoperative diet	Solid food is prohibited 12 h before surgery, no drinking water 2 h before surgery,	Fasting 12 h before surgery
Perioperative gastric tube	No gastric tube	Gastric tube
Intraoperative	Accurate operation, less bleeding	Normal operation
Intraoperative subcutaneous drainage tube	Multi-side holes silicone tube	No drainage tube
Intraoperative drainage tube	Soft silicone tube	Hard double pipe
postoperative analgesia	Multi-mode analgesia reducing the dose of Morphine	Multi-mode analgesia reducing the dose of morphine
Postoperative activities	Encourage activity	Optional activity
Postoperative eating	Drink water 6 h after surgery	Drink water after 3 days

### Discharge criteria

Taking semi-liquid or general food and no infusion can meet their own needs, getting out of bed with the help of others, The patient and his family members are willing to leave the hospital, If the patient and family members are not willing to leave the hospital, Because we have a cheaper hospital bill, We can't force patients out of the hospital, We judge and record the time when the patient may be discharged from the hospital.

### Data collection and analysis

Intraoperative hemorrhage, Postoperative anal exhaust time, The number of Wound fat liquefaction and wound dressing changing, Wound healing time, Postoperative length of stay, readmission rate, complication were recorded.

### Statistical method

All data were statistically analyzed by SPSS 19.0, Measurement data were expressed by means ± standard deviation, Adopted *T* test; *X*^2^ test was used to compare the counting data, *P* < 0.05 was considered statistically significant.

## Results

Intraoperative blood loss: ERAS group (40.57 ± 6.32 ml),CT group (83.19 ± 11.17 ml); Postoperative anal exhausting time: ERAS group (3.12 ± 0.57) days, CT group (3.88 ± 0.66) days; Number of dressing changes after surgery: ERAS group (3.72 ± 0.75), CT group(9.34 ± 1.31); Wound healing time: ERAS group (11.71 ± 0.85)days, CT group (15.50 ± 3.68)days; Postoperative length of stay: ERAS group (18.72 ± 6.96)days, CT group (19.42 ± 10.44)days; Intraoperative blood loss、Number of dressing changes after surgery、Wound healing time、 Postoperative anal exhaust time were compared in two groups, *P* < 0.05; Other indicators were compared between the two groups, *P* > 0.05 (As shown in Table 3).

**Table 3:** ERAS group and CT group postoperative statistical index

Postoperative statistical index	ERAS group	CT group	*P* value
Intraoperative blood loss(ml)	40.57 ± 6.32	83.19 ± 11.17	0.000
Postoperative anal exhaust time(day)	3.12 ± 0.57	3.88 ± 0.66	0.000
Number of dressing changes after surgery	3.72 ± 0.75	9.34 ± 1.31	0.000
Wound healing time(day)	11.71 ± 0.85	15.50 ± 3.68	0.000
Postoperative length of stay(day)	18.72 ± 6.96	19.42 ± 10.44	0.653
Cases of pulmonary infection	3	5	0.491
anastomotic fistula cases	1	3	0.360
readmission rates	0	0	
Internal jugular vein catheter infection	2	5	0.246
intraperitoneal infection	0	3	0.118
Wound fat liquefaction	2	12	0.005
death case	0	1	0.493

### Complications

Internal jugular vein catheter infection: ERAS group 2 cases, CT group 5 case; intraperitoneal infection: ERAS group 0 cases, CT group 3 case; Wound fat liquefaction: ERAS group 2 cases, CT group 12 case; pulmonary infection: ERAS group 3 cases, CT group 5 case; death case: ERAS group 0 cases, CT group 1 case; Number of anastomotic fistula cases: ERAS group 1 case, CT group 3 case, These patients were treated conservatively with double cannula flushing, The fistula healed smoothly; Comparison of readmission rates: Both groups are 0, There were significant differences in fat liquefaction between the two groups, *P* < 0.05, Other indicators were compared between the two groups, *P* > 0.05(As shown in Table 3).

## Discussion

In the 1990s, enhanced recovery after surgery (ERAS) or fast track surgery strategy was initiated in European countries and the United States to reduce surgical stress and improve outcomes after surgery [12, 13]. Various perioperative care approaches were introduced to reduce perioperative stress responses and accelerate postoperative function recovery. Core aspects included no perioperative fasting, optimal nutrition and fluid management, decreased use of tubes, optimizing pain control, and early mobilization [14, 15]. Recent meta-analyses of evidence-based studies have indicated that a reduction in the length of hospital stay and postoperative complications was achieved following ERAS implementation in the context of elective colorectal surgery, without an increase in readmission rate [16, 17].

The ERAS protocol is a model of perioperative care for patients undergoing different types of major surgeries [18]. Such protocols consist of pre-, intra-, and postoperative interventions, with the aim of minimizing surgery-related stress and promoting faster restoration of homeostasis. Several perioperative measures have proven to reduce morbidity and hospital stay in patients undergoing colorectal surgery [19]. ERAS programs streamline such interventions as a perioperative pathway leading to lower complication rates and healthcare cost reduction [20–23].

According to the characteristics of the elderly, ERAS group in this article adopted modified rapid recovery measures during perioperative period, The intraoperative blood loss was significantly reduced compared with the control group, With advances in surgical techniques、better surgical instruments(Mainly ultrasonic knife), Operating fine, Anatomical accuracy, Reducing the amount of additional tissue damage and bleeding, The stimulation to the patient is correspondingly reduced; Postoperative anal exhaust time in the ERAS group was significantly shortened compared with that in the control group, Conventional treatment lacks effective ideas and methods to relieve stress and complications and promote postoperative recovery of patients, suggesting that appropriate rapid rehabilitation measures can also significantly promote the recovery of gastrointestinal function in elderly patients.

In industrialised countries, major complications (i.e. those that are potentially life-threatening and require hospitalisation and therapeutic intervention) occur in over 25% of inpatient surgical procedures [24]. In the United States (US) alone, surgical site infections (SSIs) account for 36% of all health care-associated infections, which are a major cause of morbidity, putting 8 million US patients at risk for developing an SSI annually [25, 26].In open wounds, negative pressure therapy helps promote a wound-healing environment by reducing oedema, removing infectious materials and promoting perfusion and granulation tissue formation [27–29].

SSI represents a major health burden to patients as well when considering that SSI after major surgery has been associated with a doubling in the risk of postoperative mortality as well as increased likelihood of hospital readmission and need for ICU care [30]. Shen (2017) previously reported the results of a Phase II randomized controlled trial using negative-pressure wound therapy (NPWT) in an attempt to decrease SSI in patients undergoing laparotomy for various abdominal malignancies [31]. Negative pressure wound therapy is a device placed at the time of wound closure to promote healing by primary intention using suction (negative pressure) [32].

In the ERAS group of this paper, soft silicone tube with multiple side holes was placed subcutaneously for drainage, Poked from the lower end of the wound, The drainage tube is connected with the negative pressure ball, It is simpler and more practical than the negative pressure suction device mentioned above, It plays the role of NPWT, draining out the fat liquefy ooze, avoiding subcutaneous effusion, promoting wound healing, It do not affect the activities of patients, Postoperative wound fat liquefaction in ERAS group was significantly reduced, The postoperative number of wound dressing changes in ERAS group were less than that in the control group, The wound healing time in ERAS group were significantly shorter than that in the control group, Further hints: A drainage tube was placed under the skin of the wound during the operation (NPWT), To reduce the number of wound fat liquefaction and postoperative wound dressing and promote wound healing has an obvious effect, Correspondingly, it also has the effect of reducing stress and promoting recovery.

Soft silicone tube was indwelling in the abdominal cavity during the operation(replacing the hard double cannula in the conventional treatment group), without affecting patient activity and causing pain, It can drain fluid and blood in the abdominal cavity, with observing whether the anastomotic site is abnormal, If anastomotic fistula is present, Flushing and drainage through a silicone tube, The anastomotic fistula is healed by conservative treatment, Avoiding the possibility of a second or even third operation, Each operation can be deadly for older patients and It also extended the length of hospital stay and increased hospital expenses, Abdominal drainage tube placement is necessary for colorectal surgery in elderly patients; Other authors [32] suggest that preoperative intestinal preparation and intraoperative indwelling of an abdominal drainage tube can reduce the consequences of intestinal fistula without affecting the therapeutic effect of ERAS.For elderly or high-risk patients, intraoperative abdominal drainage tube should not be completely abandoned, ERAS should not be absolutely untubed.

There was no significant difference in postoperative pulmonary infection and postoperative hospitalization time between the two groups, It doesn't show the advantage of a quick recovery, It may be related to the number of cases, It may also be related to the degree of implementation of rapid rehabilitation measures; That is to say, the degree to which patients and their family members actively implement rapid recovery measures will affect the effect of rapid recovery.

There was no significant difference in postoperative anastomotic fistula between the two groups, It is suggested that appropriate rapid rehabilitation measures do not increase the incidence of anastomotic fistula, Early postoperative drinking water and liquid diet can promote intestinal peristalsis, It is beneficial to reduce the atrophy of disused intestinal mucosa and prevent the translocation of intestinal bacteria, but is not obvious adverse effect on the anastomosis.

Among the elderly patients, ERAS is even more rare, because there are many medical diseases in the elderly patients, ERAS is applied in elderly patients with many difficulties, and unreasonable application may cause complications, which is not conducive to patients' recovery, ERAS schemes for elderly patients should be different from those for general patients, and ERAS schemes suitable for elderly patients are urgently needed, Evidence-based ERAS protocols have since then been published and practiced in a multitude of surgical procedures. These include pancreaticoduodenectomy, colorectal surgery, and bariatric surgery, to name a few. An understanding of the most commonly employed strategies with positive outcomes is essential for practitioners to design new ERAS protocols or pick appropriate interventions for a patient’s individualized ERAS plan [33].

All the patients in the paper were over 70 years old and the physiological characteristics of the elderly patients were considered, perioperative ERAS scheme adopts the principle of individualization, preoperative half amount of bowel preparation getting rid of accumulated bowel stools, no drinking water 2 h before surgery; With regard to bowel preparation, mechanical bowel preparation (MBP) plus oral antibiotic bowel preparation (OBP) prior to colorectal surgery is associated with reduced complication rates [34]. In previous ERAS guidelines in colon [35] and rectum [36] surgery, given the universal use of systemic antibiotic prophylaxis, the recommendation has been to avoid the use of mechanical bowel preparation (MBP) in colonic surgery but that it may be advantageous in rectal surgery. The rationale behind this is to avoid preoperative dehydration, electrolyte disturbance and discomfort with no clinical gain for the patient [37].

Aiming at old people patient to have constipation mostly, intestinal tract has accumulated defecate; In this paper, Elderly patients in ERAS group and control group were treated with compound polyethylene glycol electrolyte powder, One box with 1000 ml drinking water, 1–2 boxes according to the defecation condition of each patient in ERAS group, Pull out of the mushy stool or water stool, do not need to pull too much; The preparation of the intestinal tract is accompanied by oral rehydration and electrolytes, so as not to cause dehydration and electrolyte disruption, The excretion of accumulated stool is conducive to the recovery of intestinal function.

## Conclusion

For elder patients with colorectal cancer, Modified ERAS is safe and effective, The implementation of ERAS should adopt the principle of individuation, Appropriate intestinal preparation before operation, It is beneficial to observe the occurrence and treatment of anastomotic fistula by placing a soft silicone tube in the abdominal cavity during operation, NPWT accelerates healing of wound.

## Data Availability

For data on this article, please contact the corresponding author if necessary.

## Abbreviations

ERAS: Enhanced recovery after surgery.
CT: Conventional treatment.
NPWT: Negative-pressure wound therapy.
SSIs: Surgical site infections
